# Teachers’ Personality, Perfectionism, and Self-Efficacy as Predictors for Coping Strategies Based on Personal Resources

**DOI:** 10.3389/fpsyg.2021.751930

**Published:** 2021-11-02

**Authors:** Elena Mirela Samfira, Ramona Paloş

**Affiliations:** ^1^Teacher Training Department, Banat University of Agricultural Sciences and Veterinary Medicine from Timişoara, Timişoara, Romania; ^2^Department of Psychology, West University of Timişoara, Timişoara, Romania

**Keywords:** pre-service teachers, proactive coping strategies, personality traits, perfectionism, self-efficacy

## Abstract

Many psychological constructs as personality, perfectionism, and self-efficacy have been identified to have a strong contribution to teachers’ coping strategies, but how these variables collectively predict different types of coping has received little attention. The present study aimed to explore the personal resources (personality traits, perfectionistic strivings, and self-efficacy) which predict teachers’ proactive coping strategies. The sample study consisted of 284 pre-service teachers, with ages ranging from 18 to 34years old (*M*=19.9; *SD*=2.1). Four hierarchical multiple regression analyses were conducted separately for every proactive coping strategy based on personal resources as criterion variables. Results showed that conscientiousness and openness were predictors for all four coping strategies based on personal resources (proactive, reflective, strategic planning, and preventive coping), extraversion and neuroticism predicted only proactive coping strategies, and agreeableness did not predict any kind of these coping strategies. Planfulness was a predictor for reflective, strategic planning, and preventive coping strategies; striving for excellence predicted only proactive coping, and organization was a predictor only for reflective coping strategies. Self-efficacy predicted the first three proactive coping strategies but preventive coping. Because coping strategies can be learned, knowing what personal resources may help teachers to cope with stressful situations inside and outside the school, could be organized training programs to improve activity and well-being in the teaching profession.

## Introduction

Teaching is one of the most stressful professions (Ryan et al., 2017), personal and professional responsibilities representing real challenges for most of the teachers. The way that teachers experience stress depends on the interaction between their personality traits, the skills they have developed, the values they are guided by, and the context that generated the stressful situation (Antoniou et al., 2013). To cope with all of these internal or external demands, they need to constantly invest cognitive and behavioral effort (Lazarus and Folkman, 1984). The adopted coping strategies are closely related to stressors from their teaching activity (e.g., pupil misbehavior, workload, lack of professional recognition and support, time pressure, and lack of benefits – Mintz, 2007; Collie et al., 2012; Santoro, 2018). Thus, the way they anticipate and approach the stressors or the difficult situations, and how they are motivated to overcome them, can be better explained by the perspective of the proactive coping theory framework (Greenglass et al., 1999; Schwarzer and Taubert, 2002). Focused on goal-oriented and adaptive strategies, proactive coping is important because emphasizes future, possible stressful situations that have not yet happened, and individuals may be better prepared to deal with them (Greenglass and Fiksenbaum, 2009; Ersen and Bilgiç, 2018). Moreover, in proactive coping, the highlighting is on developing strategies and resources to cope with challenges, and also on increasing the potential and opportunities for personal growth (Straud et al., 2015; Drummond and Brough, 2016).

Proactive coping has not been sufficiently studied, despite its relationship to many positive outcomes (Hambrick and McCord, 2010; Carlander and Johansson, 2020; Serrano et al., 2021). For instance, teachers’ coping strategies used in the educational environment were intensively investigated (Kahn et al., 2012; Gustems-Carnicer et al., 2019; Pogere et al., 2019; Stapleton et al., 2020), but only few studies take into consideration how these strategies are influenced by personality (David and Quintão, 2012; Antoniou and Mitsopoulou, 2017), positive perfectionism (Stoeber and Rennert, 2008), self-efficacy (Shen, 2009; Verešová and Malá, 2012), or by a combination of these variables from the perspective of proactive coping theory (Innes and Kitto, 1989; Greenglass et al., 1999; Straud et al., 2015; Drummond and Brough, 2016).

Understanding the relationships between personality and coping strategies is important, especially in training pre-service teachers (Jang et al., 2007). Selecting appropriate strategies can help future teachers overcome stressful classroom situations and see them as challenges rather than difficulties, thus facilitating their adaptation to the specific demands of the profession (Gustems-Carnicer et al., 2020). Hambrick and McCord (2010) stated that people who deal with stressful situations proactively are more likely to have specific personality traits that allow them successfully coping. Straud et al. (2015) found that proactive coping is positively predicted by conscientiousness, openness, and extraversion, while preventive coping is positively predicted by conscientiousness and openness, and negatively by neuroticism. Perfectionism, as a personality disposition to impose standards that demand high performance and achievements (Dunn et al., 2006), is linked to coping strategies as well, although there are only a few studies on this topic. For example, Aiken (2008) showed that self-oriented perfectionism, as an adaptive dimension, is positively correlated to proactive coping. Also, Jowett et al. (2016) sustained that perfectionistic strivings are best characterized by proactive coping strategies when confronting stress. Self-efficacy, the people’s belief in their capacity to mobilize all the resources to accomplish actions (Bandura, 1997), is another variable with great impact on coping strategies used by teachers. Thus, previous studies found that self-efficient teachers tend to frequently use proactive coping strategies based on personal resources (i.e., proactive, reflective, strategic, and preventive), highlighting the importance of self-efficacy in coping with a stressful situation in classrooms (Verešová and Malá, 2012; Akpochafo, 2014; Fathi et al., 2021).

Although each of these variables was investigated, there is no research on how a combination of personality traits, positive facets of perfectionism, and self-efficacy predict pre-service teachers’ proactive coping based on personal resources (i.e., proactive, reflective, strategic, and preventive coping strategies). Furthermore, only two of the strategies have got more attention – proactive and preventive strategies (Drummond and Brough, 2016; Ersen and Bilgiç, 2018). For this research, we chose to use all four strategies based on personal resources: proactive, reflective, strategic, and preventive strategies, because they can give us information about how people use their resources for self-regulation and goal attainment. Also, because proactive coping strategies can be learned and depend on the situation (Carlander and Johansson, 2020) they could be helpful in training pre-service teachers to cope with the demands of their future job, increase their engagement, and reduce the level of stress and burnout (Parker and Martin, 2009; Antoniou et al., 2013). Consequently, to fill this gap, the present paper aimed *to investigate the relationship between personality traits, positive facets of perfectionism (striving for excellence, organization, and planfulness), and self-efficacy, and how these variables predict different types of proactive coping based on personal resources (proactive, reflective, strategic, and preventive coping strategies), in pre-service teachers*.

Many studies emphasized those traditional coping strategies focused on problems or emotions, but the proactive coping approach brings a new perspective and moves the interest on how to anticipate stressful situations which are not assessed as threats, and how to be prepared to face things that have not occurred yet (Schwarzer and Taubert, 2002; Greenglass and Fiksenbaum, 2009). The four investigated proactive coping strategies are based on personal resources that people mobilized to deal with these challenges. Exploring variables that can predict these coping strategies, our results contribute to both theory and educational practice. Thus, from the theoretical perspective, they expand the knowledge of the relationships between teachers’ resources and the coping strategies they can use to meet workplace challenges. From the practical point of view, this information becomes useful in the development of programs that can help teachers to improve their coping strategies’ repertoire and build up resources to successfully manage their relationships and teaching activity.

### Coping Strategies

Coping involves “efforts to control harmful, threatening, or challenging conditions that occur when a routine or habitual response is not readily available” (Renard and Snelgar, 2015, p. 169). It is essential to understand how people face difficult situations and how they manage their cognitive and behavioral resources to adapt to stressful circumstances (Lazarus and Folkman, 1984; Renard and Snelgar, 2015). Folkman and Lazarus (1980) made the distinction between two kinds of coping responses: problem-focused coping and emotion-focused coping. Greenglass et al. (1999) developed a positive multidimensional approach of coping with seven types of strategies, based on an individual’s resourcefulness, vision, and responsibility, emphasizing the difference between proactive coping and the other types of coping. They considered that proactive people know how to use personal and social resources, have a vision of success, set goals and strive to achieve them, use positive emotional strategies, and take responsibility for making things happen (Greenglass et al., 1999). Moreover, proactive coping can be considered as proactive behavior because is future-oriented, people trying to anticipate potential stressors and to prevent them from occurring (Greenglass and Fiksenbaum, 2009). Thus, the first type of strategy is proactive coping – linked to the behaviors and cognitions associated with self-regulatory goal attainment; reflective coping characterizes people who analyze a variety of possible behavioral alternatives to see which of them are effective; strategic planning strategies are focused on breaking extensive tasks into manageable components; preventive coping is centered on how to be prepared for anticipated stressors or possible threats in the future; instrumental support seeking focused on getting advice, feedback, or information from people which are part of the own social network; emotional support seeking is concentrated on how to regulate emotions through the disclosure of ones’ feeling to others; and avoidance coping referring to avoiding action in a demanding situation (Greenglass et al., 1999; Greenglass, 2002). The first four coping strategies (i.e., proactive, reflective, preventive, and strategic) can be considered positive coping strategies based on personal resources, while instrumental and emotional support seeking are based on social resources that people seek in others. Avoidance coping strategies are based on personal resources that people do not invest in solving stressful situations (Greenglass, 2002). Hence, analyzing the characteristics of the proactive, reflective, preventive, and strategic coping strategies, it can be considered they are largely equivalent to problem-focused strategies, based on the interpretation of the stressful situation, the anticipation of the consequences of the aversive situation, and individuals’ view of their ability to face the stressors (Légeron, 1993; Tielemans et al., 2015). The main difference between the proactive perspective and the perspective proposed by Folkman and Lazarus (1980) is that proactive coping becomes”goal management instead of risk management” (Greenglass and Fiksenbaum, 2009, p. 30). So, people are “proactive,” take initiatives, develop their resources, and put effort to achieve their goals. They are not just “reactive” to a stressful event that is happening in the present (Schwarzer and Taubert, 2002).

### Personality

The Five-Factor Model of Personality (FFM) is one of the most known models for conceptualizing personality traits (McCrae and Costa, 1987). The five dimensions described are conscientiousness – as representing a strong sense of aim and a high level of aspirations and achievements; openness – related to the individual’s need for variety, curiosity, novelty, and change; extraversion – emphasizing a preference to appreciate companionship and to seek social stimulation; agreeableness – linked to the willingness to cooperate and to be a compassionate person; and neuroticism – as representing a tendency to experience sadness, guilt, hopelessness at the negative pole, and emotional maturity, self-confidence, and the ability to cope with a stressful situation, at the positive pole (i.e., emotional stability as low neuroticism; Goldberg, 1990; McCrae and Costa, 2008).

The personality structure may have an impact both on the stressors’ level and the way people cope with different stressing situations (Lee-Baggley et al., 2005; Connor-Smith and Flachsbart, 2007; Reevy and Frydenberg, 2011; Otero-López et al., 2021). The results of previous research on the impact of innate dispositions on specific coping strategies are mixed. For example, McCrae and Costa (1986) postulated that our favorite coping style can be derived directly from some specific personality traits (e.g., extraversion and neuroticism), while Carver et al. (1989) were in opposition with this statement. Kardum and Krapić (2001) considered that personality factors are involved in the process of coping, and it is essential to understand how they may influence people’s choice of certain strategies (Hambrick and McCord, 2010). For instance, the authors suggested that a combination of a high level in conscientiousness (especially achievement-striving), extraversion (especially cheerfulness), and agreeableness (especially altruism), and a low level in neuroticism (especially depression) are a set of characteristics that emphasize good coping ability (Hambrick and McCord, 2010). Although the literature is scarce regarding proactive coping (i.e., proactive, reflective, strategic, and preventive) and its relationships with personality traits (Ersen and Bilgiç, 2018), some studies showed a negative correlation between neuroticism and proactive and preventive coping (Drummond and Brough, 2016). Straud et al. (2015) found that conscientiousness, openness, and extraversion are positive predictors for proactive coping, while conscientiousness and openness positively predict preventive coping. Neuroticism was found to negatively predict proactive coping strategies. According to study results of Serrano et al. (2021), proactive coping is positively predicted by extraversion, conscientiousness, and openness, and negatively by neuroticism and agreeableness. In the case of preventive coping, neuroticism and agreeableness are negative predictors, whereas conscientiousness and openness are positive predictors.

Because the relationship between personality traits, coping strategies, and environment influences the level of classroom’ stressful situations (Antoniou et al., 2013), some authors highlighted that personality traits play a significant role in who choose to become a teacher (Paloş and Gunaru, 2017; Kell, 2019). For example, some principals select those candidates with high scores on specific personality traits, such as caring (equivalent to agreeableness), creativity (equivalent to openness to experience), enthusiasm (equivalent to extraversion), and motivation (equivalent to conscientiousness; Engel and Finch, 2015). Based on the above results, we assumed that:

*H1*: The personality traits positively relate to coping strategies based on personal resources.

### Perfectionistic Strivings

Considered as multidimensional and multifaceted personality traits (Stoeber and Rennert, 2008; Hill et al., 2016), perfectionism consists of having high standards of performance, overly-critical evaluation about one’s behaviors, fears and doubts of one’s actions, and over-concern about other’s evaluation and criticism (Frost et al., 1990; Hewitt and Flett, 1991). Hill et al. (2004) described perfectionism as an eight-dimensional construct: organization, high standards for others, striving for excellence, planfulness, concern over mistakes, need for approval, parental pressure, and rumination. These eight dimensions are merged in two domains facets (Hill et al., 2004): conscientious perfectionism (organization, planfulness, striving for excellence, and high standards for others) and self-evaluative perfectionism (rumination, need for approval, concern over mistakes, and parental pressure). Other authors identified two types of perfectionism: a normal, healthy, or adaptive one – with positive attributes and outcomes, and another one being considered neurotic, unhealthy, or maladaptive – associated with negative attributes and outcomes (Frost et al., 1993; Stoeber and Otto, 2006).

Originally called positive striving perfectionism and maladaptive evaluative concern perfectionism (Frost et al., 1993), these dimensions became perfectionistic strivings and perfectionistic concerns (Stoeber and Otto, 2006). Perfectionistic strivings select those facets of perfectionism considered normal, healthy, or adaptive from perfectionistic behaviors characterized by high personal standards, self-oriented perfectionism, and striving for perfection (Stoeber and Otto, 2006; Stoeber et al., 2020). The perfectionistic concern includes those facets of perfectionism considered unhealthy, neurotic, or maladaptive, and is characterized by concern over mistakes, doubts about actions, negative reaction to imperfections, and fear of other’ negative evaluation. Striving for perfection, because of the correlations with positive characteristics, processes, and outcomes (Stoeber and Otto, 2006), is recommended to be seen as a “healthy pursuit of excellence” (Shafran et al., 2002). People high in perfectionistic strivings are very motivated to succeed, regulate their emotions very well, and highly engage in proactive coping strategies (Hinterman et al., 2012; Smith et al., 2015; de la Fuente et al., 2020a). Rice and Lapsley (2001) found that individuals with a high level of perfectionistic strivings tend to use active coping strategies more often than maladaptive perfectionists and non-perfectionists when they try to deal with the stressor or to reduce its effect. Also, Stoeber and Rennert (2008) showed that teachers’ striving for perfection positively predict active coping strategies, focused on how to plan efforts to overcome the stressful event, to learn the lesson from the experience, and to see it as a challenge.

Perfectionistic strivings were measured by taking into consideration organization and personal standards (Blankstein and Dunkley, 2002), but in this study, we decided to combine organization, planfulness, and striving for excellence to assess perfectionistic strivings, as representing the positive perfectionism based only on personal resources. According to the above results, we formulated the second hypothesis:

*H2*: The perfectionistic strivings positively relate to coping strategies based on personal resources.

### Self-Efficacy

Self-efficacy is defined as “people’s judgments of their capabilities to organize and execute courses of action required to attain designated types of performances” (Bandura, 1986, p. 391). Self-efficacious individuals are more involved in their tasks and more tenacious in the front of obstacles, self-efficacy making the difference in how individuals think, feel, and behave (Bandura, 1997). When people believe that they are capable to accomplish specific tasks, they are willing to initiate more difficult tasks than less efficacious people (Locke and Latham, 2006). Also, being confident in their abilities, people think that particular behaviors could lead to their success so, they will keep them constantly and will be more tenacious in front of impasses (Zimmerman, 2000).

The level of self-efficacy is strongly related to individuals’ perception of stress (Bandura, 1995) and is seen as a personal resource for coping with all types of job stress (e.g., work overload; Bandura, 1997). Devonport and Lane (2006) found that self-efficacy represents one of the most important non-contextual factors which affect coping strategies. Moreover, Greenglass (2002) sustain that it is necessary to feel competent to manage a stressful situation, because “self-efficacy predicts future behavior” (Herman et al., 2018, p. 91). Self-efficacy and coping strategies represent two of the most studied personal resources in managing stressful situations (Freire et al., 2020). Thus, although there is not much research on the proactive coping framework proposed by Greenglass et al. (1999), previous research showed that teachers’ self-efficacy positively correlates with proactive coping strategies (Vernon et al., 2009; Nizielski et al., 2013). Verešová and Malá (2012) reported a positive association between teachers’ self-efficacy and all four types of proactive coping strategies (i.e., proactive, reflective, strategic, and preventive). Also, Shen (2009) found that teachers with high levels of general self-efficacy tend to use more active coping strategies than passive coping strategies. It seems that the level of self-efficacy increases the prevalence of applying active coping strategies to manage classrooms challenges (Betoret and Artiga, 2010). Contrary, Chan (2008) found that pre-service teachers’ self-efficacy did not predict active coping strategy.

Generally, self-efficacious teachers cope better with stressful situations and tension at school (Pajares, 2006; Fathi et al., 2021), are more protected from job strain (Schwarzer and Hallum, 2008), are more satisfied with their job (Perera and John, 2020), and feel less stressed and neurotic (Jamil et al., 2012; Verešová and Malá, 2012). They are more sociable and confident in their abilities to become successful teachers (Jamil et al., 2012), face fewer difficulties in managing students’ misbehaviors (Caprara et al., 2003), being more open to change – when they perceive less external pressure (Barni et al., 2019). Freire et al. (2020) found that self-efficient teachers showed more flexibility in their coping strategies. Based on the above arguments, the third hypothesis postulated that:

*H3*: Self-efficacy positively relates to coping strategies based on personal resources, after controlling for the effect of personality traits and perfectionistic strivings.

## Materials and Methods

### Participants

The convenience sample consisted of 284 Romanian pre-service teachers from the Banat University of Agricultural Sciences and Veterinary Medicine. The university specializes in life sciences and veterinary medicine and prepares students in six distinct faculties. The participants were selected from all students of the university who were enrolled in the Educational Psychology course (66.5% females and 33.5% males; 60.6% from the urban area and 39.4% from rural area), in the first year of their teachers’ preparation program. The age of the participants ranged between 18 and 34 years (*M*=19.9; *SD*=2.1), and their participation was voluntary. Also, they received extra credit in the Educational Psychology course.

### Procedures

During Educational Psychology, which is one of the courses that students attend in their first year of teachers training, the first author presented the aim of the research and invited students to participate. All the subjects who accepted to participate in this study signed an Informed Consent Form, according to the Ethical standards in research with human subjects, and they were assured that could give up the study whenever they wanted, without any negative consequences. The four questionnaires were administered individually, in a paper-and-pencil format, between October 2019 and February 2020. The study was ruled inside the university campus, and all the procedures were following the ethical standards of the institutional research committee, being under the 1964 Helsinki declaration and its later amendments or comparable ethical standards.

### Instruments

The first part of the questionnaires contained demographic information: age, gender, and type of locality.

*Coping strategies* based on personal resources were assessed with *Proactive Coping Inventory* (PCI; Greenglass et al., 1999). PCI is a 55-item measure, using a four-point Likert scale, from (1) not at all true to (4) completely true. The questionnaire consists of seven scales (types of coping): proactive, reflective, strategic, and preventive coping, instrumental and emotional support seeking, and avoidance coping. In this research, only the scale based on personal resources were used: proactive, reflective, strategic, and preventive coping. Alpha’s Cronbach for the entire scale was 0.89, and for the subscales ranged between 0.62 and 0.82.

*Personality* traits were measured using *The International Personality Item Pool* (IPIP-50; Goldberg, 1992; Romanian version, IPIP-50, by Rusu et al., 2012), a 50-item questionnaire assessing the five dimensions of personality: agreeableness, conscientiousness, emotional stability (low neuroticism), extraversion, and openness to experience. The questionnaire uses a five-point Likert scale, from (1) strongly disagree to (5) strongly agree. For the entire scale Cronbach’s alpha was 0.88, and for the scales ranged from 0.69 to 0.81.

*Perfectionism* was assessed with *The Perfectionism Inventory Scale* (PI; Hill et al., 2004), validated for the Romanian teachers’ population by Samfira and Maricuțoiu (2021). The scale has 59-item and assessed eight dimensions of perfectionism: concern over mistakes, high standards for others, need for approval, organization, planfulness, perceived parental pressure, rumination, and striving for excellence. The participants need to give their answers on a five-point Likert scale, from (1) strongly disagree to (5) strongly agree. For this research, only the components of perfectionistic strivings, the positive dimension of perfectionism (Stoeber and Otto, 2006; Stoeber et al., 2020) were used as personal resources, respectively *organization* (the tendency to be neat and orderly), *planfulness* (the tendency to plan and to deliberate over decisions), and *striving for excellence* (the tendency to pursue perfects results and high standards; Hill et al., 2004). Alpha Cronbach for the entire questionnaire was 0.91 and ranged from 0.71 to 0.93 for the sub-scales.

*Self-efficacy* was measured with the *General Self-Efficacy* scale (GSE; Schwarzer and Jerusalem, 1995), a 10-item questionnaire, developed to assess a general sense of perceived self-efficacy (Schwarzer and Jerusalem, 1995). The scale uses a four-point Likert, from (1) not at all true to (4) exactly true. The internal consistency of the scale was 0.75.

### Data Analysis

The statistical software package SPSS 23.0 was used to analyze the data. For testing the hypotheses, four hierarchical multiple regression analyses were conducted separately for every coping strategy based on personal resources as criterion variables, and personality traits and perfectionism strivings as predictor variables. Personality traits were introduced in the first step because they are considered very stable; in the second step were introduced perfectionism strivings dimensions, also considered stable dimensions, and the general self-efficacy was introduced in the last step, being considered strongly related with individuals’ perception of stress (Bandura, 1995).

## Results

Descriptive statistics and the correlation matrix between all the research variables are presented in Table 1.

**Table 1:** Correlation matrix between study variables.

Variables	*M*	*SD*	1	2	3	4	5	6	7	8	9	10	11	12	13
Proactive coping	46.20	5.72	*(0.82)*												
Reflective coping	33.89	5.38	0.44^**^	*(0.78)*											
Strategic planning	11.81	2.32	0.50^**^	0.54^**^	*(0.62)*										
Preventive coping	30.57	5.21	0.40^**^	0.49^**^	0.60^**^	*(0.80)*									
Extraversion	33.39	7.92	0.44^**^	0.12^*^	0.19^**^	0.11	*(0.83)*								
Agreeableness	39.86	5.08	0.37^**^	0.26^**^	0.27^**^	0.21^**^	40^**^	*(0.69)*							
Conscientiousness	36.92	6.81	0.47^**^	0.26^**^	0.46^**^	0.44^**^	0.21^**^	0.38^**^	*(0.77)*						
Emotional stability	28.10	7.62	0.33^**^	0.13^*^	0.17^**^	0.13^*^	0.35^**^	0.21^**^	0.26^**^	*(0.81)*					
Openness	37.77	5.46	0.48^**^	0.33^**^	0.31^**^	0.28^**^	0.32^**^	0.35^**^	0.28^**^	0.18^**^	*(0.74)*				
Organization	3.77	0.98	0.35^**^	0.18^**^	0.39^**^	0.39^**^	0.12^*^	0.27^**^	0.79^**^	0.14^*^	0.17^**^	*(0.93)*			
Planfulness	3.71	0.72	0.33^**^	0.42^**^	0.50^**^	0.53^**^	−0.00	0.23^**^	0.45^**^	0.11	0.27^**^	0.53^**^	*(0.79)*		
Striving for excellence	3.64	0.83	0.37^**^	0.14^*^	0.26^**^	0.31^**^	0.14^*^	0.23^**^	0.39^**^	−0.07	0.24^**^	0.46^**^	0.40^**^	*(0.81)*	
General self-efficacy	33.25	3.87	0.64^**^	0.36^**^	0.43^**^	0.32^**^	0.24^**^	0.27^**^	41^**^	0.27^**^	0.37^**^	0.31^**^	0.31^**^	0.27^**^	*(0.75)*

*N*=284; Proactive, reflective, strategic planning, and preventive coping=proactive coping strategies based on personal resources; Extraversion, Agreeableness, Conscientiousness, Emotional stability, and Openness=personality traits; and Organization, Planfulness, and Striving for excellence=Perfectionistic strivings. Values of the internal consistency alphas are displayed in italic in the diagonal.

* *p* <0.05;

** *p* <0.01.

As can be seen, the results partially support the first hypothesis which stated that *the personality traits positively relate to coping strategies based on personal resources*. Agreeableness correlated positively and significantly with proactive coping (*r*=0.37, *p* < 0.001), reflective coping (*r*=0.26, *p* < 0.001), strategic planning (*r*=0.27, *p* < 0.001), and preventive coping (*r*=0.21, *p* < 0.001). Conscientiousness correlated positively and significantly with proactive coping (*r*=0.47, *p* < 0.001), reflective coping (*r*=0.26, *p* < 0.001), strategic planning (*r*=0.46, *p* < 0.001), and preventive coping (*r*=0.44, *p* < 0.001). Openness correlated positively and significantly with proactive coping (*r*=0.48, *p* < 0.001), reflective coping (*r*=0.33, *p* < 0.001), strategic planning (*r*=0.31, *p* < 0.001), and preventive coping (*r*=0.28, *p* < 0.001). Emotional stability correlated positively and significantly with proactive coping (*r*=0.33, *p* < 0.001), reflective coping (*r*=0.13, *p* < 0.001), strategic planning (*r*=0.17, *p* < 0.001), and preventive coping (*r*=0.13, *p* < 0.001). Extraversion correlated positively and significantly only with proactive coping (*r*=0.44, *p* < 0.001), reflective coping (*r*=0.12, *p* < 0.05), and strategic planning (*r*=0.19, *p* < 0.001). Thus, all the five personality dimensions’ correlate positively and significantly to proactive, reflective, and strategic coping strategies, while the preventive coping strategies show significant positive relationships only to four of the personality dimensions (i.e., agreeableness, conscientiousness, emotional stability, and openness).

The second hypothesis: *the perfectionistic strivings positively relate to coping strategies based on personal resources*, received full statistical support. Organization correlated positively and significantly with proactive coping (*r*=0.35, *p* < 0.001), reflective coping (*r*=0.18, *p* < 0.001), strategic planning (*r*=0.39, *p* < 0.001), and preventive coping (*r*=0.39, *p* < 0.001). Planfulness correlated positively and significantly with proactive coping (*r*=0.33, *p* < 0.001), reflective coping (*r*=0.42, *p* < 0.001), strategic planning (*r*=0.50, *p* < 0.001), and preventive coping (*r*=0.53, *p* < 0.001). Striving for excellence correlated positively and significantly with proactive coping (*r*=0.37, *p* < 0.001), reflective coping (*r*=0.14, *p* < 0.05), strategic planning (*r*=0.26, *p* < 0.001), and preventive coping (*r*=0.31, *p* < 0.001). Therefore, all the positive facets of perfectionism, organization, planfulness, and striving for excellence, positively correlate to proactive, reflective, strategic planning, and preventive coping strategies.

For the third hypothesis: *self-efficacy positively relates to coping strategies based on personal resources, after controlling for the effect of personality traits and perfectionistic strivings*, Table 2 presents the results of the regression analysis with the four dependent variables. Regarding the *proactive coping strategies* as a criterion variable, in the first step personality traits accounted for 43% of the variance, and the model was significant [*F*(5, 278)=41.97; *p*<0.000] with extraversion (*β*=0.23; *p*<0.000), conscientiousness (*β*=0.29; *p*<0.000), emotional stability (*β*=0.11; *p*<0.02), and openness (*β*=0.28; *p*<0.000) as significant predictors. By adding in the second step of the regression model, the perfectionistic strivings dimensions and controlling the influence of the personality traits, the predictive value of the second model increases to 46.7% (Δ*R*^2^=0.036) with only the striving for excellence dimension as a significant predictor (*β*=0.18; *p*<0.001). In step 3, general self-efficacy was added to the regression model (*β*=0.39; *p*=001) and explained 11.3% of the additional variance [Δ*R*^2^=0.113; *p*=0.001; *F*(9, 274)=41.95; *p*=0.001], after controlling the influence of the personality traits and the perfectionistic strivings dimensions. The final model that includes all predictors explained teachers’ use of proactive coping strategies at a rate of 57.9% (*R*^2^=0.579).

**Table 2:** Hierarchical regression analysis predicting the four types of proactive coping strategies (i.e., proactive, reflective, strategic, and preventive coping strategies).

Variables	Proactive coping			Reflective coping			Strategic planning			Preventive coping		
	*R* ^2^	Δ*R*^2^	*ß*	*R* ^2^	Δ*R*^2^	*ß*	*R* ^2^	Δ*R*^2^	*ß*	*R* ^2^	Δ*R*^2^	*ß*
*Step 1*	0.430^**^	0.430^**^		0.155^**^	0.155^**^		0.251^**^	0.251^**^		0.224^**^	0.224^**^	
Extraversion			0.231^**^			−0.061			0.027			−0.035
Agreeableness			0.050			0.126			0.047			0.008
Conscientiousness			0.295^**^			0.138^*^			0.381^**^			0.396^**^
Emotional stability			0.112^*^			0.044			0.026			0.003
Openness			0.282^**^			0.265^**^			0.173^**^			0.181^**^
*Step 2*	0.467^**^	0.036^**^		0.253^**^	0.098^**^		0.347^**^	0.096^**^		0.349^**^	0.125^**^	
Extraversion			0.237^**^			0.018			0.099			0.039
Agreeableness			0.028			0.095			0.015			−0.031
Conscientiousness			0.264^**^			0.158			0.303^**^			0.300^**^
Emotional stability			0.145^**^			0.012			0.012			0.003
Openness			0.232^**^			0.189^**^			0.098			0.088
Organization			−0.094			−0.188^*^			−0.083			−0.109
Planfulness			0.106			0.395^**^			0.378^**^			0.411^**^
Striving for excellence			0.181^**^			−0.059			−0.011			0.061
*Step 3*	0.579^**^	0.113^**^		0.282^**^	0.029^**^		0.378^**^	0.031^**^		0.353	0.004	
Extraversion			0.210^**^			0.004			0.085			0.033
Agreeableness			0.026			0.094			0.014			−0.031
Conscientiousness			0.155^*^			0.103			0.245^**^			0.279^**^
Emotional stability			0.089^*^			−0.017			−0.018			−0.008
Openness			0.151^**^			0.148^*^			0.056			0.072
Organization			−0.058			−0.170			−0.064			−0.102
Planfulness			0.057			0.370^**^			0.352^**^			0.401^**^
Striving for excellence			0.139^**^			−0.080			−0.033			0.053
General self-efficacy			0.397^**^			0.200^**^			0.209^**^			0.076

*N*=284; Proactive, reflective, strategic planning, and preventive coping=proactive coping strategies based on personal resources; Extraversion, Agreeableness, Conscientiousness, Emotional stability, and Openness=personality traits; Organization, Planfulness, and Striving for excellence=Perfectionistic strivings.

* *p*<0.05;

** *p*<0.01.

For the second regression analysis with the *reflective coping strategies* as a criterion measure, in the first step personality traits accounted for 15.5% of the variance, and the model was significant [*F*(5, 278)=10.21; *p*=0.001] with only conscientiousness (*β*=0.13; *p*<0.05) and openness (*β*=0.26; *p*<0.000) as significant predictors. By adding in the second step of the regression model the perfectionistic strivings dimensions and controlling the influence of the personality traits, the predictive value of the second model increases to 25.3% (Δ*R*^2^=0.098) with organization dimension as a significant negative predictor (*β*=0.18; *p*<0.05), and planfulness as a significant positive predictor (*β*=0.39; *p*<0.001). In step 3, general self-efficacy was added to the regression model (*β*=0.20; *p*<0.001) and explained 2.9% of the additional variance [Δ*R*^2^=0.029; *p*=0.000; *F*(9, 274)=11.94; *p*=0.001], after controlling the influence of the personality traits and the perfectionistic strivings dimensions. The final model that includes all predictors explained teachers’ use of reflective coping strategies at a rate of 28.2% (*R*^2^=0.282).

Regarding the *strategic planning coping strategies* as a criterion variable, in the first step personality traits accounted for 25.1% of the variance, and the model was significant [*F*(5, 278)=18.62; *p*<0.000] with conscientiousness (*β*=0.38; *p*<0.001), and openness (*β*=0.17; *p*<0.003) as significant predictors. By adding in the second step of the regression model the perfectionistic strivings dimensions and controlling the influence of the personality traits, the predictive value of the second model increases to 34.7% (Δ*R*^2^=0.096) with only the planfulness dimension as a significant predictor (*β*=0.37; *p*<0.001). In step 3, general self-efficacy was added to the regression model (*β*=0.20; *p*<0.001) and explained 3.1% of the additional variance [Δ*R*^2^=0.031; *p*=0.001; *F*(9, 274)=18.53; *p*=0.001] after controlling the influence of the personality traits and perfectionistic strivings dimensions. The final model that includes all predictors explained teachers’ use of strategic planning coping strategies at a rate of 37.8% (*R*^2^=0.378).

For the last regression analysis with *preventive coping strategies*, in the first step personality traits accounted for 22.4% of the variance, and the model was significant [*F*(5, 278)=16.04; *p*<0.001] with conscientiousness (*β*=0.39; *p*<0.001), and openness (*β*=0.18; *p*<0.002) as significant predictors. By adding in the second step of the regression model the perfectionistic strivings dimensions and controlling the influence of the personality traits, the predictive value of the second model increases to 34.9% (Δ*R*^2^=0.125) with only the planfulness dimension as a significant predictor (*β*=0.41; *p*<0.001). In step 3, general self-efficacy did not significantly add to the explained variance [*F*(9, 274)=16.60; ns].

These results indicated that general self-efficacy positively relates to proactive, reflective, and strategic coping strategies, and were not related to preventive coping strategies after controlling the personality traits and perfectionistic strivings dimensions. Thus, the third hypothesis received only partial statistical support.

## Discussion

The present study aimed to investigate the relationship between personality traits, positive facets of perfectionism (i.e., striving for excellence, organization, and planfulness), and self-efficacy, and how these variables predict different types of proactive coping based on personal resources (i.e., proactive, reflective, strategic, and preventive coping strategies), in pre-service teachers.

The results partially confirmed the *first hypothesis*. All the five personality traits correlate positively and significantly with three of the coping strategies based on personal resources (proactive, reflective, and strategic), while only four of the personality dimensions (agreeableness, conscientiousness, emotional stability, and openness) correlate positively and significantly with the preventive coping strategies. Despite these relationships, when the personality traits were introduced into the regression analysis, things changed. Thus, only conscientiousness and openness predicted all the four coping strategies based on personal resources. Extraversion and emotional stability were significant positive predictors only for proactive coping strategies, while agreeableness was not a predictor for any of the coping strategies based on personal resources.

Conscientiousness and openness positively predict proactive, reflective, strategic, and preventive coping strategies, in the case of pre-service teachers. Conscientious people can plan future actions, are hardworking, persevering, responsible, organized (Barrick and Mount, 1991), and use self-control strategies more efficiently and for a long time (Russell et al., 2017). Openness to experience is linked to open-minded and intellectual curiosity, behavioral flexibility (Costa and McCrae, 1992), and coping planning (Watson and Hubbard, 1996). All of these are attributes that can help future teachers to anticipate and identify stressors related to the teaching profession (i.e., preventive coping), to be prepared to deal with them (Greenglass et al., 1999; Renard and Snelgar, 2015). When change occurs, people with a high level of conscientiousness focus their attention and effort on understanding the situation and finding new ways of doing things. Perseverance helps them in searching for information that can help them to make good decisions (Le Pine et al., 2000). After a non-threatening evaluation of the new circumstances (Schwarzer and Taubert, 2002), they can develop a constructive perspective of success, mobilizing internal resources to achieve setting goals (i.e., proactive coping; Greenglass et al., 1999). Reflective and strategic planning coping strategies help teachers to analyze possible alternatives and solutions trying to see which of them are more efficient in specific situations and to break the tasks into smaller, more manageable pieces to succeed. Openness to experience becomes essential because open people are willing to experience new things, be creative, and find new ways of solving problems. Moreover, it seems that people with a high level of openness make better decisions after a routine situation has changed (Le Pine et al., 2000). So, pre-service teachers who are conscientious and open to experiences are more inclined to adopt all these proactive coping strategies to cope with classroom stressful situations. They experience less stress and gained more academic achievement (Gustems-Carnicer et al., 2019). Conscientiousness and openness to experience are considered essential predictors for the decision-making process after the change in context (Le Pine et al., 2000), and can become important personal resources for pre-service teachers in adopting proactive coping strategies in their future profession.

Extraversion and emotional stability significantly predicted proactive coping strategies. People with high scores for extraversion tend to be assertive, sociable, and energetic, experiencing positive emotions (Costa and McCrae, 1992). Previous research showed that extraversion is a predictor for experiencing positive life events (Magnus et al., 1993), and a positive predictor for problem-focused coping strategies (de la Fuente et al., 2020b). Individuals with high scores at neuroticism are likely to be anxious, insecure, with poor impulse control (Barrick and Mount, 1991), while those with low scores are emotional-stable and relaxed (i.e., emotional stability; Costa and McCrae, 1992). Neuroticism is considered a predictor for individuals to experience negative life events (Magnus et al., 1993), and a negative predictor for positive coping strategies (Straud et al., 2015; de la Fuente et al., 2020b). Teachers with a high level of extraversion and low level of neuroticism can keep their positive vision of the situation based on the positive appraisal of the stressors, have enough energy to persist in coping efforts by using personal resources to deal with everyday challenging situations in school, and to achieve their goals (Greenglass et al., 1999; Connor-Smith and Flachsbart, 2007; Straud et al., 2015). Torgersen (1995) spoke about a “complicated type” category of people with a high level of extraversion, neuroticism, and conscientiousness. He sustained that a combination of high levels of extraversion and conscientiousness could counterbalance the negative effects of the high level of neuroticism. So, individuals with complicated types were not so distressed person as we expect, due to their high neuroticism, their coping profile being considered extremely well-functioning (Vollrath and Torgersen, 2000).

Our results are quite similar to some studies which identified personality traits as predictors for proactive coping strategies, but other findings are not consistent with the existing literature. For instance, Straud et al. (2015) found that proactive coping strategies are positively predicted by conscientiousness, openness, and extraversion, and negatively by neuroticism, while Serrano et al. (2021) reported also the agreeableness dimension as a negative predictor, alongside neuroticism. A possible explanation could be linked to the instrument used to assess personality traits, respectively neuroticism (i.e., BFI or NEO-PI-R) instead of emotional stability (i.e., IPIP-50). Regarding preventive coping strategies, our results are in line with those reported by Straud et al. (2015), but different from the findings of Serrano et al. (2021) who found agreeableness and neuroticism dimensions as negative predictors. In our study, there were positive relationships between agreeableness and all four types of coping strategies based on personal resources, but this personality trait was not a predictor for any of these coping strategies, which is in line with Straud et al. (2015) findings.

The results confirmed the *second hypothesis*, too. Organization, planfulness, and striving for excellence positively correlated with all four coping strategies based on personal resources. Once introduced into regression analysis, organization, as a personal resource of perfectionistic strivings, became a negative predictor for reflective coping. Reflective coping strategies are based on analysis of a variety of alternatives to behave and see which of them are effective (Greenglass et al., 1999; Greenglass, 2002). To compare and evaluate the solutions need to be organized, but, being too organized can be an impediment to reflect on identifying as many alternatives as possible. Planfulness, as the “tendency to plan and to deliberate over decisions” (Hill et al., 2004, p. 83) was a positive predictor for three coping strategies based on personal resources (i.e., reflective, strategic planning, and preventive coping). This tendency to carefully plan and make an informed decision helps teachers cope with everyday educational situations. Planfulness was not a predictor for proactive coping, which makes us think that teachers’ tendency to plan everything may lead them to some rigidity and inability to have the vision to act proactively. Allinder (1994) stated that organized and planful teachers are more experienced in instructional practices, have stronger beliefs in their ability to teach, and are more fair and firm in dealing with their students, and also with stressful situations they encounter. Despite the significant correlations, striving for excellence was a positive predictor only for proactive coping. As a component of the perfectionistic strivings, this type of coping involves behaviors and cognitions associated with self-regulatory goal attainment. Teachers’ striving for excellence, considered as an invested effort to be “the personal best” (Brown, 2011, p. 57), needs to be seen as a way to improve the performance and not as a competition (Brown, 2011). For example, Hill et al. (2004) findings suggested that pre-service teachers who have the “tendency to pursue perfect results and high standards” (p. 83) are more likely to adopt a positive strategy based on personal resources: to work hard to identify and analyze stressors, problems, and resources, to identify appropriate solutions, and to implement them, to achieve excellent outcomes, in the professional and personal context.

The findings of the present study partially confirmed the *third hypothesis*. Thus, self-efficacy, as a personal resource, was positively related to all four positive coping strategies but predicted only three of them – proactive, reflective, and strategic coping. Considered an essential characteristic in dealing with a demanding and stressful situation (Freire et al., 2020), teachers with a high level of self-efficacy are very confident in their ability to mobilize resources, evaluate alternatives, and follow those actions needed to efficiently cope with stressors. However, self-efficacy was not a predictor of preventive coping strategies. One possible explanation could be that, through the preventive coping strategies, people try, based on their previous knowledge and experience, to anticipate a potentially stressful event to be prepared to deal with it (Greenglass et al., 1999; Renard and Snelgar, 2015). So, having strong efficacy beliefs about their capacity to succeed in specific situations, teachers appraise stressful events as challenging not as threatening, which can influence their choices, effort, and time invested in facing obstacles (Bandura, 2000). These findings are in line with Social Cognitive Theory of Bandura (2001) which claims that individuals with a high level of self-efficacy trust their abilities to respond effectively to the various stimuli from the environment. Also, Devonport and Lane (2006) sustained that self-efficacious people could pay attention to the opportunities and challenges, which may help them to cope with stressful events. It seems that teachers with high levels of self-efficacy tend to use proactive, problem-focused, or adaptive coping strategies (Carver et al., 1989; Shen, 2009; Vernon et al., 2009).

### Theoretical and Practical Implications

The findings of the present study bring new insights on the pre-service teachers’ proactive coping strategies based on personal resources, having both theoretical and practical implications. Hence, our findings add knowledge regarding the positive facets of perfectionism (striving for excellence, organization, and planfulness) as predictors for coping strategies. Sometimes, perfectionistic teachers are seen negatively, being often considered dissatisfied and always working to achieve perfection (Stoeber and Rennert, 2008). But a clear distinction needs to be made between teachers with negative perfectionism and those who have high standards, who are well organized and want to achieve excellence (i.e., positive perfectionism). Positive perfectionistic teachers can analyze and select the best solutions to cope with stressful and challenging situations from an educational context. Knowing the coping strategies and personal resources used by pre-service teachers in managing conflicts and tensions specific to the teaching career helps identify and develop agency in teachers (Pillen et al., 2013; Yayli, 2017). A teacher “equipped” with a strong self can resist any kind of school-related pressure (Foucault, 1980).

Practical implications are related to training programs use to help pre-service teachers to identify their coping style and to develop proactive coping strategies to improve the capacity to manage challenging situations and, finally, to experience well-being in the teaching profession (Shen, 2009; Cook et al., 2017; de la Fuente et al., 2020a; Stapleton et al., 2020). These programs could include activities to increase self-efficacy, strive for excellence in teaching activities, and to become aware that openness and conscientiousness are important predictors for proactive coping strategies based on personal resources. Understanding the relationships between personality traits, positive facets of perfectionism, and self-efficacy as predictors for adopting proactive coping strategies, pre-service teachers can avoid unproductive or negative coping strategies, and, consequently, prevent anxiety, stress, depression, and teachers’ attrition (; Murray-Harvey et al., 2000; Hartwick and Kang, 2013; MacIntyre et al., 2020; Martínez et al., 2020). In the face of a challenging and stressful situation, teachers may ask for advice to alter the context (to create better conditions for teaching and learning) or may remain silent without taking any action, considering that they are not able to change anything (Admiraal et al., 2000). Another practical implication is related to the role of proactive coping strategies and personal resources in managing the possible tensions between professional requirements as a teacher and personal aspirations or possibilities (Beijaard et al., 2004). During the teachers’ training, being aware of their coping strategies and the level of personal resources, pre-service teachers have the chance to learn how to find a balance between personal and professional life to adapt to the demands of the teaching profession.

### Limits of the Study and Future Directions

The present research has some limitations as well. First, the sample was not representative of the Romanian pre-service teachers, and the findings cannot be extrapolated to the entire population of pre-service teachers from the country. A larger sample of pre-service teachers from other universities could bring more in-depth information on coping strategies based on personal resources and maybe more different and complex findings. Second, our study analyses only personality, perfectionism, and self-efficacy as predictors for proactive coping strategies. It could be useful to identify if other personal resources, such as assertiveness or a sense of humor, can boost the effect of personality traits on teachers’ proactive coping strategies. Third, the study does not allow for causal inferences regarding the relationships between the involved variables. Hence, further research is needed to understand how personality traits (including here the perfectionism as well) influence teachers’ coping strategies choice, and how pre-service teachers can be helped to develop or improve their strengths. Moreover, it would be useful for educational practice to investigate the impact of conscientiousness, emotional stability, and extraversion facets on proactive coping strategies, knowing that they are surely linked to coping (Connor-Smith and Flachsbart, 2007).

## Conclusion

By examining proactive coping strategies, personality, perfectionistic strivings, and self-efficacy, the current paper has enlarged previous research results, relevant to the predictors of teachers’ proactive coping. In the present research, pre-service teachers’ personality (openness to experience and conscientiousness), perfectionistic strivings (planfulness, striving for excellence, and organization), and self-efficacy predicted proactive coping strategies based on personal resources (proactive, reflective, strategic planning, and preventive coping). These results have theoretical and practical implications for teacher training programs’ responsible and school principals, in improving activity and wellbeing in the teaching profession.

## Data Availability Statement

The raw data supporting the conclusions of this article will be made available by the authors, without undue reservation.

## Ethics Statement

Ethical review and approval was not required for the study on human participants in accordance with the local legislation and institutional requirements. The patients/participants provided their written informed consent to participate in this study.

## Author Contributions

ES has chosen the topic, coordinated the collection of the data, and contributed to the writing and supervision of the present manuscript. RP has contributed to the design, methodology, writing, and supervision of the present manuscript. All authors contributed to the article and approved the submitted version.

## Conflict of Interest

The authors declare that the research was conducted in the absence of any commercial or financial relationships that could be construed as a potential conflict of interest.

## Publisher’s Note

All claims expressed in this article are solely those of the authors and do not necessarily represent those of their affiliated organizations, or those of the publisher, the editors and the reviewers. Any product that may be evaluated in this article, or claim that may be made by its manufacturer, is not guaranteed or endorsed by the publisher.
